# Inhibiting autophagy potentiates the antitumor efficacy of *Euphorbia royleana* for canine mammary gland tumors

**DOI:** 10.1186/s12917-020-02408-1

**Published:** 2020-06-12

**Authors:** Yu-Ya Huang, Chia-Hung Chen, Chia-Hui Hsu, Tsun-Yung Kuo, Cheng-Chi Liu, Albert Tai-Ching Liao, Chen-Si Lin

**Affiliations:** 1 Department and Graduate Institute of Veterinary Medicine, School of Veterinary Medicine, National Taiwan University, No.1 Sec.4 Roosevelt Rd, Taipei, 10617 Taiwan; 2 Institute of Molecular Medicine and Bioengineering, National Chiao Tung University, Hsinchu City, 30068 Taiwan; 3 Department of Biotechnology and Animal Science, National Ilan University, Ilan, 26041 Taiwan; 4 Animal Cancer Center, College of Bioresources and Agriculture, National Taiwan University, Taipei, 10617 Taiwan

**Keywords:** Autophagic cell death, Apoptosis, Anti-tumor efficacy, Canine mammary tumors, Herbal medicine

## Abstract

**Background:**

Canine mammary gland tumors (cMGTs) are the most common neoplasms in intact female canines and viewed as a suitable model for studying human breast cancers. *Euphorbia royleana* has been reported to have a variety of antitumor efficacies. We have prepared the crude extracts of *E. royleana* in ethanol and hexane solvents to evaluate the anti-tumor effects for cMGT in vitro and in vivo.

**Results:**

The results showed that *E. royleana* could inhibit cell proliferation and colony formation in cMGT cells. The suppression of tumor cell growth resulted from necrosis and cell cycle arrest. Moreover, autophagy appears to play a critical role in *E. royleana*-mediated cell death by triggering cell apoptosis. The in vivo results also revealed that *E. royleana* treatment could reduce the size of solid tumors while exhibiting low toxicity in cMGT-bearing nude mice.

**Conclusions:**

The anti-tumor mechanisms of *E. royleana* were firstly verified to show it would cause autophagic cell death, apoptosis, and cell cycle arrest in canine mammary tumor cells. The in vitro and in vivo findings in the present study revealed *E. royleana* has potential anticancer effects for the treatment of canine mammary gland tumors.

## Background

Canine mammary gland tumor (cMGT) is one of the commonest tumors diagnosed in old intact female canines. The epidemiological, histopathological, and clinical characteristics as well as the biological behaviors of cMGTs in female canines are similar to human breast cancer and viewed as an excellent comparative disease model [1, 2]. Approximately 50% of the cases are diagnosed as malignant mammary carcinomas [3]. Old age, mixed breed, and large size represent risk factors for malignancy [4]. Surgical resection remains the most widely accepted treatment option for cMGTs, and adjuvant therapies such as chemotherapy, hormonal therapy, and radiation therapy are necessary; however, the cancer still recurs at a 66% rate after surgery in cMGT cases [5]. Therefore, to investigate effective therapies is significant and crucial for mammary tumor-bearing dogs.

*Euphorbia royleana* is a medicinal shrub of *Euphorbiaceae* family*.* Various species in the family have been reported to be able to inhibit cancer development [6, 7], including *E. hebecarpa* and *E. microciadia* against HeLa tumor cells, *E. osyridea* against bladder carcinoma cells, and *E. cheiradenia* against leukemia cell lines [8]. There are several ways to extract *E. royleana*, and each demonstrated different functions on tumor cells and the immune system [9]. The ethyl acetate fraction from the latex of *E. royleana* has considerable analgesic, antipyretic, anti-inflammatory, and immunosuppressive activity in animal models while its hexane fraction was proved to have antitumor effects [8, 10].

Cell death can result from necrosis, apoptosis, and autophagy; however, the role of autophagy in cancer cell death remains controversial. Autophagy appears to be tumor suppressive during cancer development but may contribute to tumor cell survival during cancer progression [11, 12]. Many studies also showed inhibiting autophagy can enhance the therapeutic benefits of various cancer therapies [13–15].

This study aims to investigate the antitumor effects and mechanisms of *E. royleana* for canine mammary tumors. The functions of autophagic inhibitors on prohibiting cancer growth with *E. royleana* are also validated to define the potential role of autophagy in cMGT development. Our in vitro and in vivo findings have revealed the administration of *E. royleana* extracts possessed an efficient tumor suppression and could be a possibly therapeutic option for canine mammary cancers.

## Results

### *E. royleana* extracts inhibited cell proliferation of cMGT cells

**Fig. 1:**
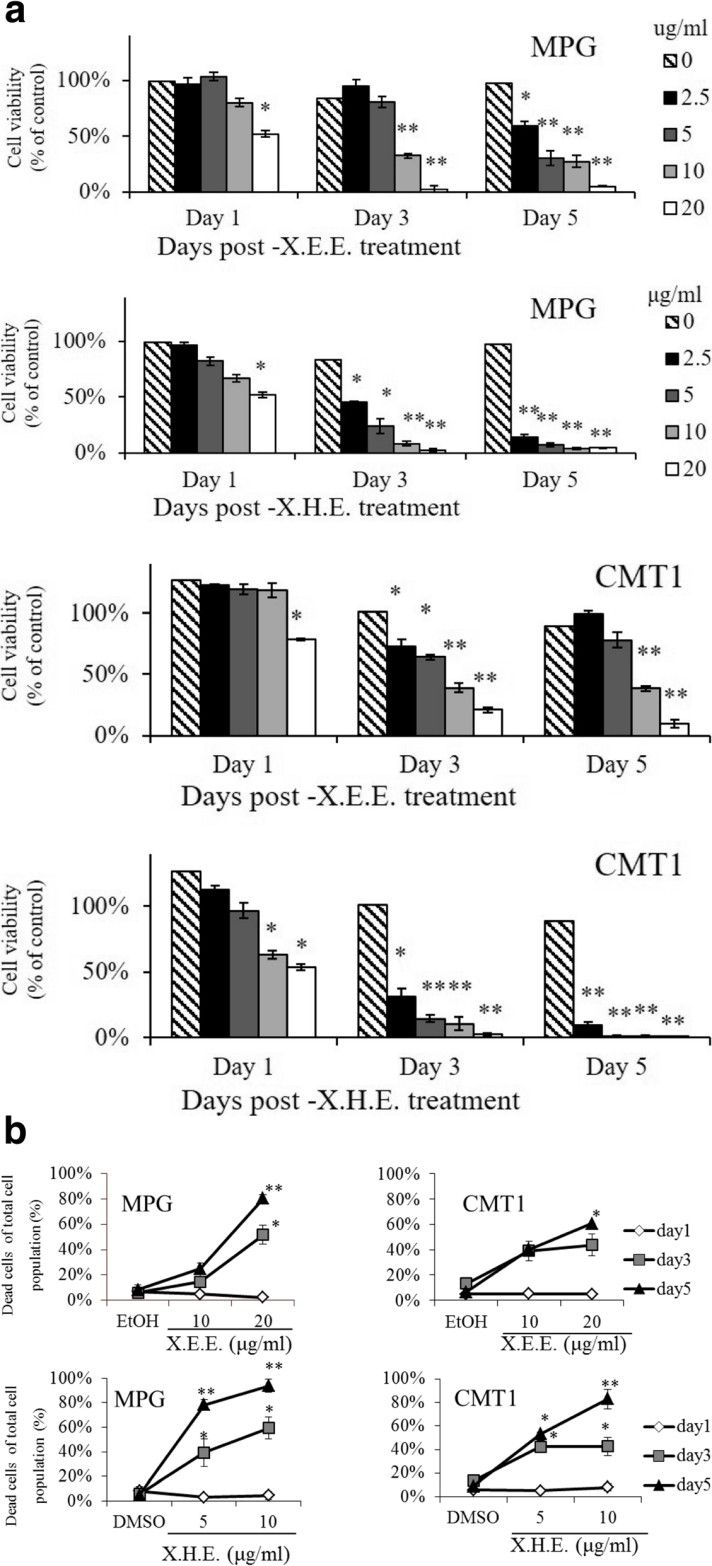
Anti-proliferative effect of *E. royleana* extracts on cMGT cells. (**a**) MPG and CMT1 cells treated with X.E.E. and X.H.E. from 2.5 to 20 μg/mL for 1, 3, and 5 days. Cell viability was detected through WST1 assay. (**b**) MPG and CMT1 cells treated with X.E.E. and X.H.E. from 5 to 20 μg/mL for 1, 3, and 5 days. Dead cells were detected by the trypan blue exclusion. Each column or point represents the means ± SDs of experiments performed using triplicate samples. *: *p* < 0.05; **: *p* < 0.01

To investigate the potential of the ethanol extract of *E. royleana* (X.E.E.) and hexane extract of *E. royleana* (X.H.E.) for cell growth inhibition of cMGTs, the antiproliferative effect of X.E.E. and X.H.E. in MPG and CMT1 cells was first measured. Cell viability was determined through WST1 assay. X.E.E. and X.H.E. inhibited cell growth in both cancer cell lines in a dose- and time-dependent manner (Fig. 1a). To determine whether the cell growth inhibition was due to cell death, we used trypan blue exclusion to find a dose- and time-dependent cell death was resulted after treating cMGT cell with X.E.E. and X.H.E.. Over 50% cell death of MPG cells treated with 20 μg/mL X.E.E. 10 μg/mL X.H.E was observed on day 3; over 50% cell death in CMT1 cells treated with 20 μg/mL X.E.E. or 10 μg/mL X.H.E. was observed on day 5 (Fig. 1b). These results demonstrated that X.E.E. and X.H.E. inhibited tumor cell growth by inducing the death of cMGT cells. The safety of *E. royleana* extracts for normal cells was subsequently validated. The treatment of normal cell lines containing both canine MDCK and non-human primate Marc145 and Vero cells with X.E.E. and X.H.E. revealed limited cytotoxicity (Table 1) at high concentrations and treatment time. The IC_50_ values implied that X.E.E and X.H.E. targeted only the tumor cells and were relatively safe for normal cells.

**Table 1:** IC_50_ values of X.E.E. and X.H.E. on cMGT cell lines, canine MDCK, and non-primate Marc145, and Vero cells

IC50 (μg/ml)^a^						
	MPG	CMT1		MDCK	Marc145	Vero
**X.E.E.**	12 ± 3.1	14 ± 5.2	**X.E.E.**	98 ± 6.7	94 ± 7.9	68 ± 9.2
**X.H.E.**	3.5 ± 1.5	4.5 ± 2.5	**X.H.E.**	36 ± 8.3	33 ± 9.2	16 ± 7.3

^a^Triplicate experiments were performed when cells were treated for 72 h with triplicate wells for each treatment

### *E. royleana* extracts reduced the colony formation of cMGT cells

**Fig. 2:**
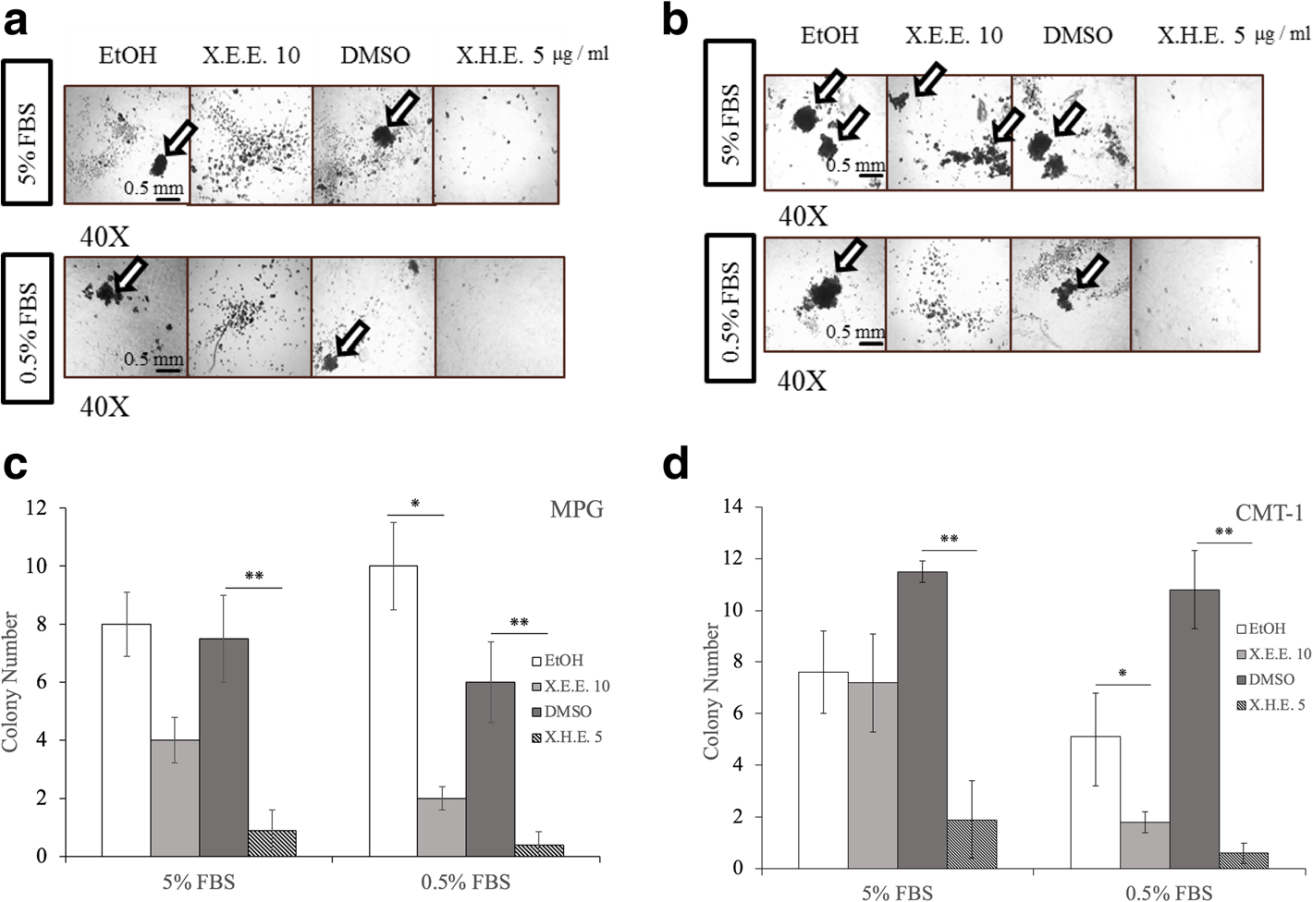
Colony formation of cMGT cell lines after exposure to different concentrations of X.E.E. and X.H.E. Microscope images reveal no apparent colony formation in (**a**) MPG cells and (**b**) CMT1 cells after incubation of X.H.E. 5 μg/mL for 14 days. Quantification of the colony number of (**c**) MPG cells and (**d**) CMT1 cells in five images is illustrated by bar charts. Each column represents the means ± SDs. *: *p* < 0.05; **: *p* < 0.01

A 3D colony formation model was next used to further validate the tumor suppression ability of *E. royleana* extracts. MPG and CMT1 cells treated with 10 μg/mL X.E.E. or 5 μg/mL X.H.E. for 14 days exhibited fewer expanding colonies compared with the untreated control cells. The colony formation of MPG cells treated with 5 μg/mL X.H.E. in 5% FBS was significantly lower compared with control cells; MPG cells treated with 10 μg/mL X.E.E. or 5 μg/mL X.H.E. in the 0.5% FBS exhibited significantly lower colony formation (Fig. 2a & c). The colony formation of CMT1 cells with or without 10 μg/mL X.E.E. or 5 μg/mL X.H.E. revealed similar results (Fig. 2b and d). Therefore, *E. royleana* extracts significantly reduced the ability of clonogenic cell survival.

### *E. royleana* extracts induced necrotic death of cMGT cells

**Fig. 3:**
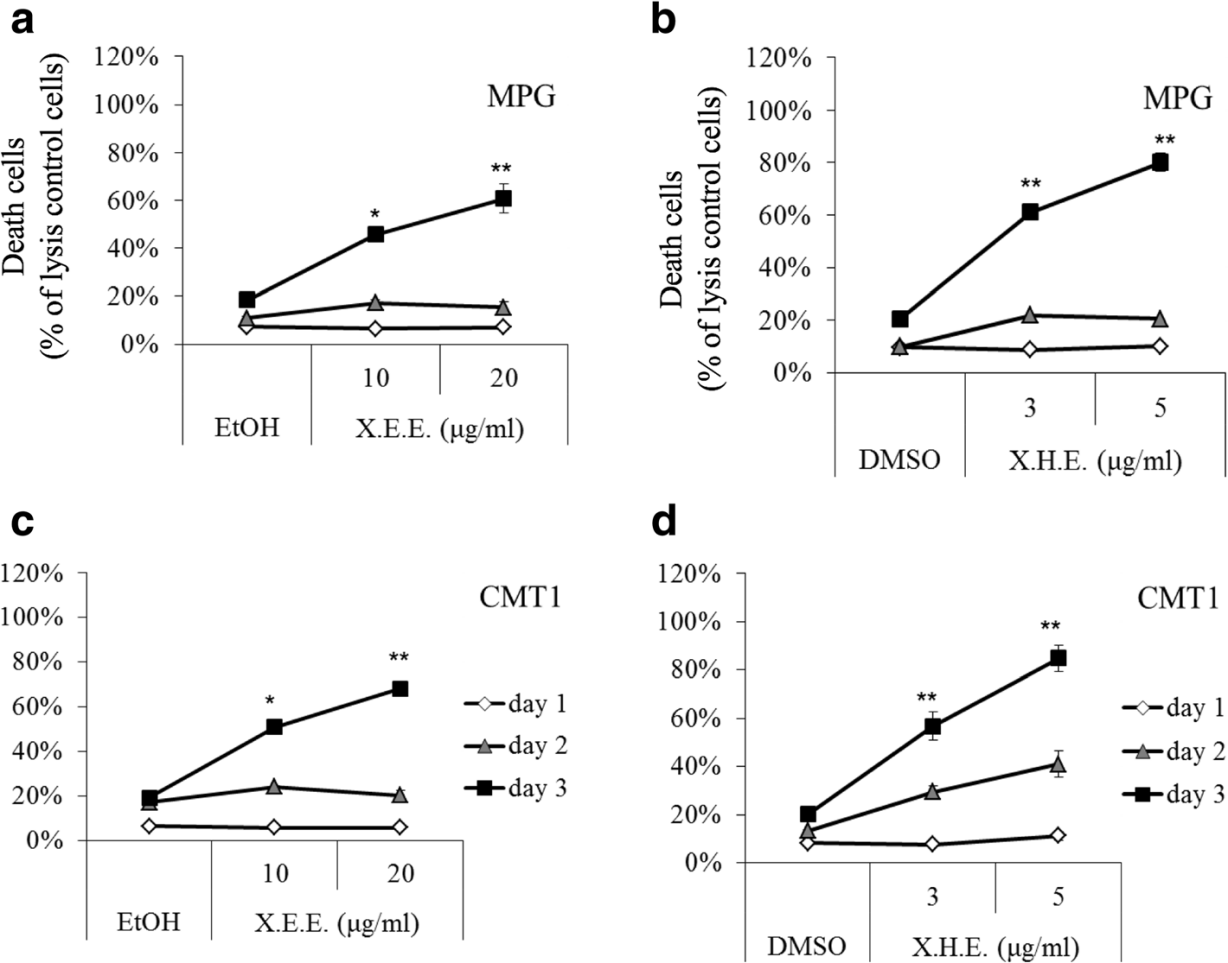
*E. royleana* induced necrotic cell death of cMGT cells. MPG cells treated with (**a**) X.E.E. and (**b**) X.H.E.; CMT1 cells treated with (**c**) X.E.E. and (**d**) X.H.E. for 1, 2, and 3 days. The amount of LDH released is expressed as a percentage of necrotic cells. Each point represents the means ± SDs of experiments performed using triplicate samples. *: *p* < 0.05; **: *p* < 0.01

X.E.E. and X.H.E. have been proved to induce the death of cMGT cells (Fig. 1). Therefore, the examination of the phenotypic consequences of cell death from *E. royleana* treatment of cMGT cells was crucial. Necrosis was detected by the release of LDH releasing assay. MPG and CMT1 cells with X.E.E. and X.H.E. elicited the release of LDH after 3 days of agonist application (Fig. 3a~d). These data demonstrated that exposure to X.E.E. and X.H.E. for 3 days could lead to highly detectable necrosis.

### *E. royleana* extracts induced cell cycle arrest of cMGT cells

**Fig. 4:**
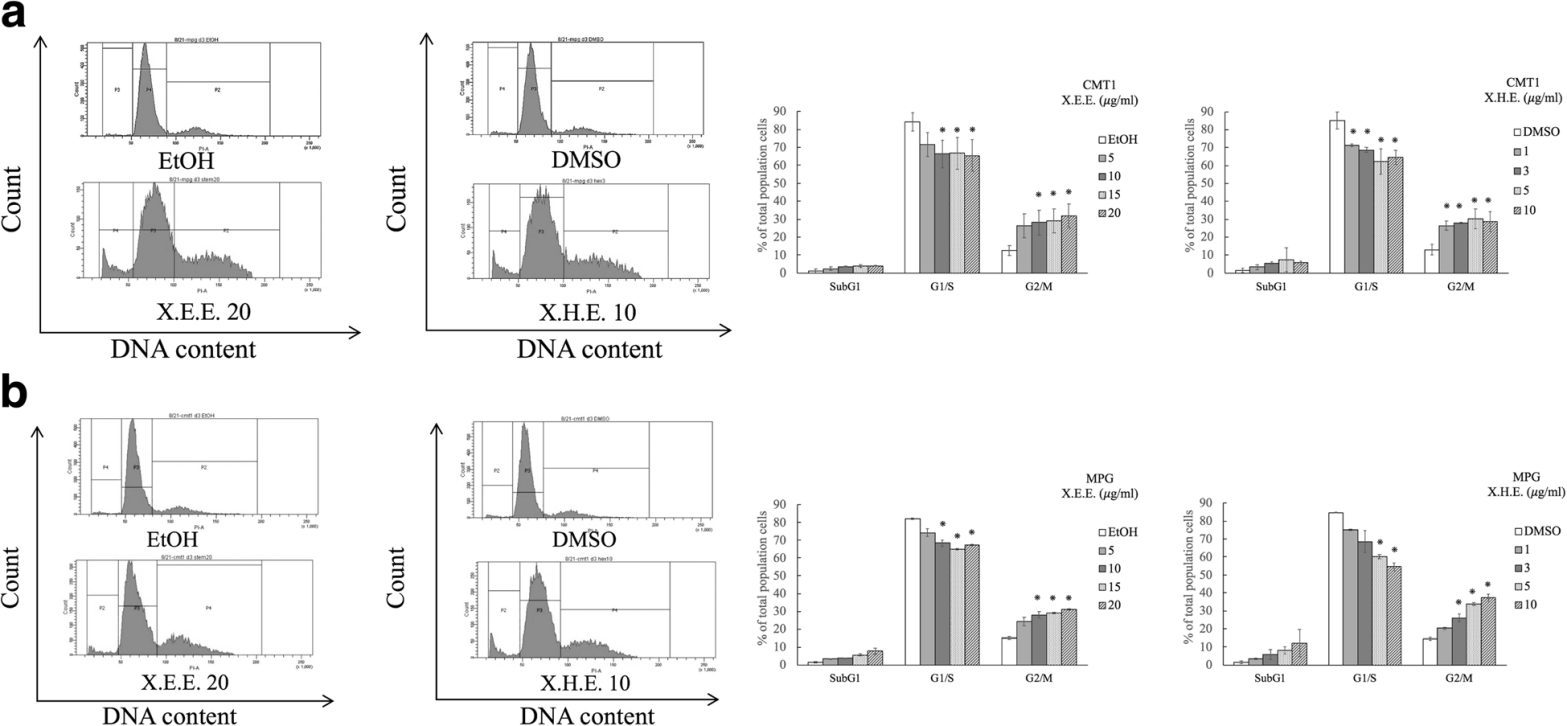
*E. royleana* caused cell cycle arrest in cMGT cells. (**a**) CMT1 and (**b**) MPG cells were treated with X.E.E. and X.H.E. for 3 days. Assay using PI staining for cell cycle analysis. The G1/S phase population decreased, and the G2/M phase population increased in a dose-dependent manner compared with control cells. Each column represents the means ± SDs of experiments performed using triplicate samples. *: *p* < 0.05; **: *p* < 0.01

**Fig. 5:**
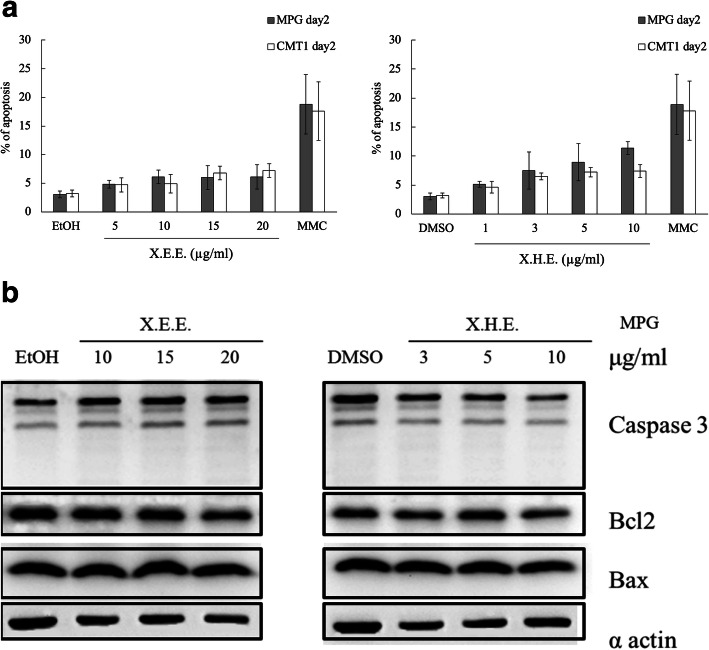
*E. royleana* extracts induced no apoptosis in the cMGT cells. (**a**) MPG and CMT1 cells treated with X.E.E. and X.H.E. for 3 days and assayed by annexin V for apoptotic analysis. Mytomycine C (5 μg/mL) was used as positive control. Each column represents the means ± SDs. (**b**) Western blotting for caspase 3, Bcl2, and Bax expression

Whether X.E.E. and X.H.E. modulate the cell cycle progression in cMGT cells was next tested. After 3 days of X.E.E. and X.H.E. treatment on MPG and CMT1 cells, the G1/S phase population decreased, and the G2/M phase increased in a dose-dependent manner compared with control cells (Fig. 4). Therefore, these results suggest that X.E.E. and X.H.E. cause cell cycle arrest at the G2/M phase in cMGT cells. Though cMGT cells treating *E. royleana* was found a significantly reduced cell number on day 1, 3 and 5 (Fig. 1), no significant apoptosis was observed for 2 days of X.E.E. and X.H.E. incubation. Analyzing these cells with annexin V assay revealed few apoptotic cells (Fig. 5a), and the levels of caspase 3, Bax (Bcl-2-associated X protein), and Bcl2 (B-cell lymphoma 2) exposed to X.E.E. and X.H.E also did not increase in MPG cells (Fig. 5b).

### Autophagy was implicated in *E. royleana*-treated cMGT cells

**Fig. 6:**
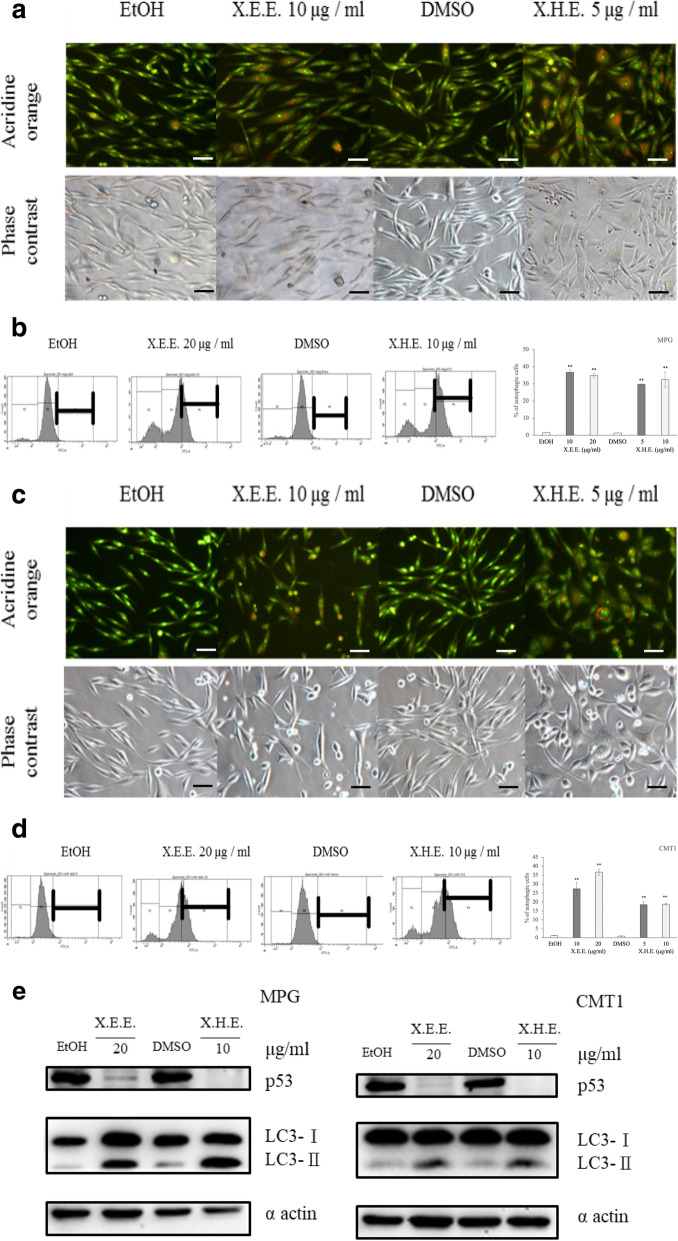
*E. royleana* induced autophagy in cMGT cells. (**a**) (**b**) MPG and (**c) (d**) CMT1 cells were treated with 10 μg/mL X.E.E. and 5 μg/mL X.H.E. for 2 days; staining with AO increased the number of orange-colored cells under the fluorescence microscope. Cells were also analyzed through flow cytometry. Each column represents the means ± SDs. (**e**) Autophagy-related marker LC3 II and cytoplasmic p53 expression were influenced by X.E.E. and X.H.E. treatment. **: *p* < 0.01

The minimal levels of apoptosis in *E. royleana* exposing cMGT cells has drawn our attention to inspect the possible role of autophagy in this condition. The formation of autophagic vesicles can be observed when the autosome fuses with lysosome via acridine orange (AO) staining. AO crosses into lysosomes (and other acidic compartments) and becomes protonated. The protonated dye stacks and stacked acridine orange emits in the red range. Acridine orange not in an acidic compartment emits as green. The results showed MPG and CMT1 cells treated with 10 μg/mL X.E.E. or 5 μg/mL X.H.E. to indicate the significant autophagic vesicle formation on day 2 (Fig. 6a and c). Quantified data by flow cytometry also showed the similar results (Fig. 6b and d). The decreased expression of p53 and increased expression of autophagosome marker LC3 (microtubule-associated light chain 3) (Fig. 6e) proved that autophagy was indeed involved in this process.

**Fig. 7:**
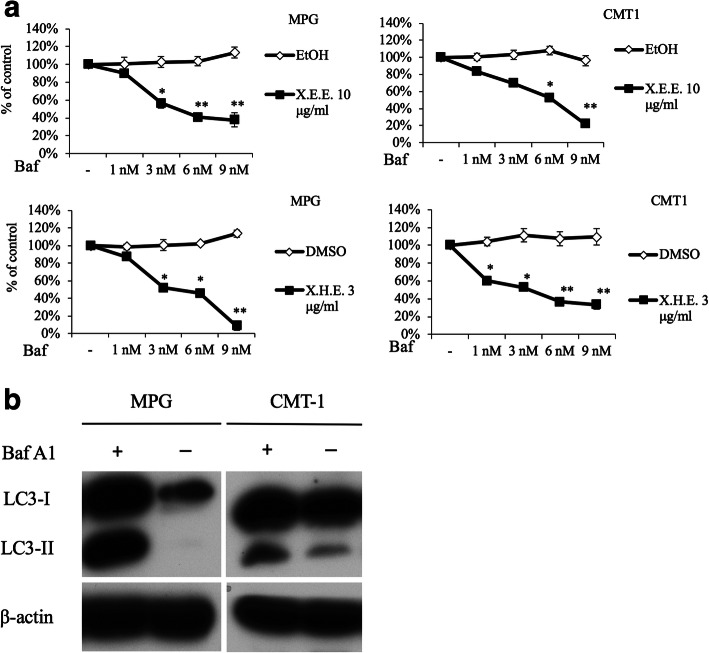
Blocking autophagy enhanced X.E.E. and X.H.E cell death. (**a**) Pre-culture cells with different concentration of autophagy inhibitor, Baf A1, then culture MPG and CMT1 cells with X.E.E. 10 μg/ml or X.H.E. 3 μg/ml for 2 days. Cell viability was determined after 2 days by WST1 assay. (**b**) The cells were treated with bafilomycin (Baf) (6 nM) for 4 h and microtubule-associated protein 1 light chain 3 (LC3-I & II) expression was measured to demonstrate the autophagy-blocking ability of Baf A1

Blocking autophagy was suggested to sensitize cancer cells to apoptosis [16]. We wondered if blocking autophagy could further enhance the antitumor effect of *E. royleana* extracts. Pretreating the autophagic inhibitor Baf as well as culturing cells with X.E.E. and X.H.E. could significantly inhibit cell growth in both cancer cell lines to reveal autophagy has rescued *E. royleana*-exposed CMT cells from even more severe cell death (Fig. 7).

### *E. royleana* inhibited tumor growth in vivo

**Fig. 8:**
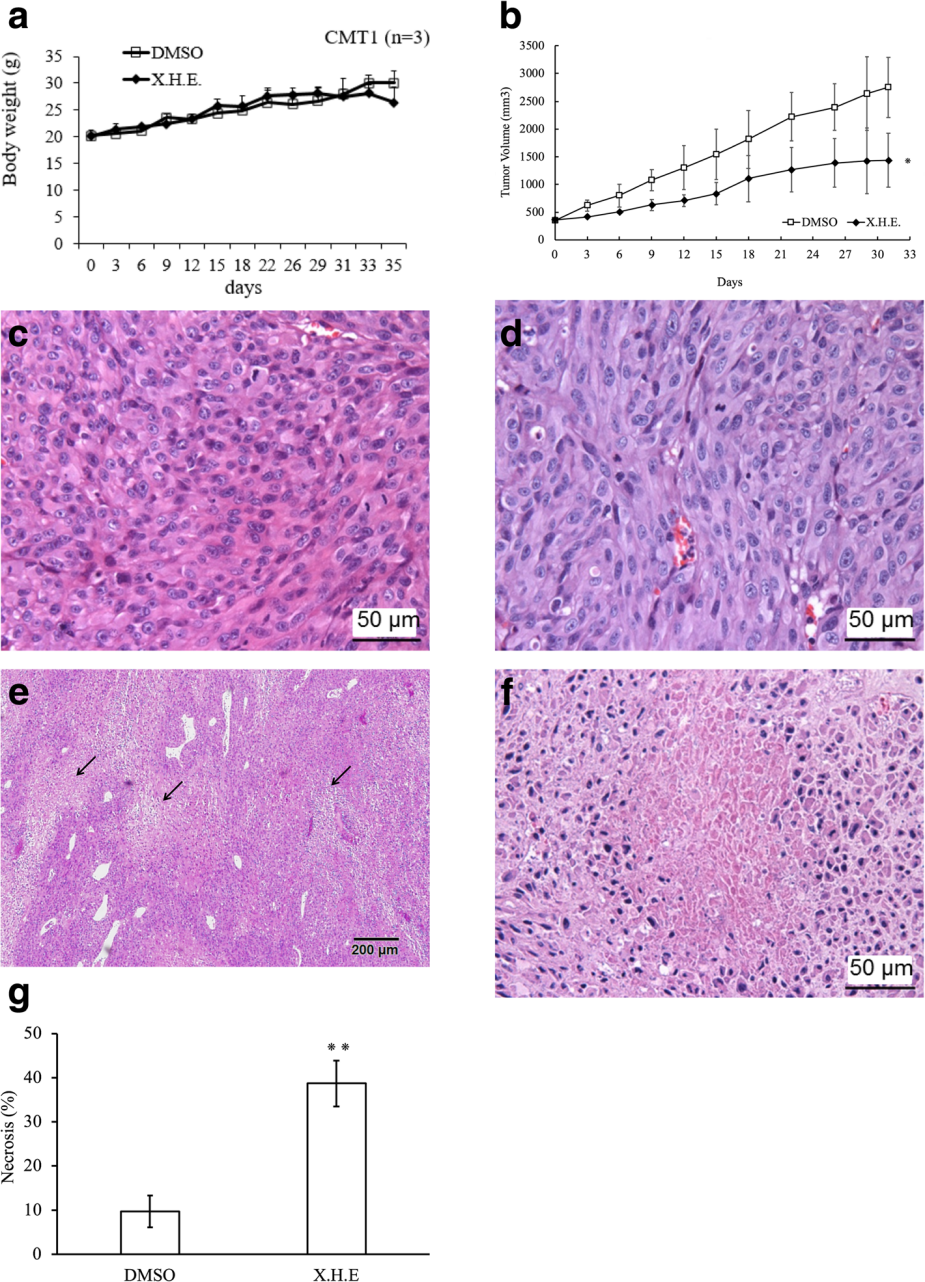
*E. royleana* inhibited the growth of xenograft cMGT. (**a**) Changes in mouse body weight. (**b**) X.H.E. inhibited the growth of tumor volumes in vivo. Euthanized CMT1-bearing nude mice after treatment with X.H.E. or vehicle-treated control group. Hematoxylin and eosin–stained tumor tissue slides were photographed. In the vehicle-treated group, (**c**) saline and (**d**) DMSO exhibited normal nuclear morphology characterized by diffuse chromatin structures. X.H.E-treated group exhibited (**e**) necrosis in tumor tissue (arrow) and (**f**) cell shrinkage, nuclear condensation, increased darkness, and fragmentation. (**g**) Quantification of necrotic area in tumors. *: *p* < 0.05; **: *p* < 0.01

To assess the effects of *E. royleana* extract on tumor growth in vivo, CMT1 cells were subcutaneously injected into nude mice. Oral administration of X.H.E. every 2–4 days did not affect the body weights of the mice (Fig. 8a); however, the tumor volumes were significantly lower in the X.H.E.-treated mice (Fig. 8b). Necrotic areas in tumor tissue were discovered, and tumor cells exhibited shrinkage, nuclear condensation, and fragmentation, whereas the vehicle-treated control group exhibited normal nuclear morphology characterized by a diffuse chromatin structure (Fig. 8c–g). These results demonstrate that X.H.E. reduces the size of solid tumors and induces tumor cell necrosis in CMT1 cell-inoculated nude mice.

## Discussion

This study demonstrated *E. royleana* extract triggered necrosis and arrested cell cycle in cMGT cells to inhibit the development of canine mammary tumors. We also found when combined with autophagy inhibitors, it could further induce apoptosis and potentiate the anti-tumor efficacy of *E. royleana.* These results indicated the extracts of *E. royleana* appear to be promising candidates for cMGT treatment.

*E. royleana* induced significant cell death in cMGT cells, we further considered the main cell death mechanisms: necrosis, apoptosis, and autophagy [17]. Because the release of LDH was highly detectable and staining with AO indicated increased prominent autophagic vesicle formation, we could confirm that necrosis and autophagy were involved in the *E. royleana* treatment of cMGT cells. Surprisingly, few annexin V–positive cells and little change in apoptosis-related proteins caspase 3, Bcl2, and Bax in cMGT cells during *E. royleana* treatment indicated that no obvious apoptosis was involved in our experimental settings.

Numerous studies have revealed the cross-talk among necrosis, apoptosis, and autophagy [18, 19]. Autophagy and apoptosis share certain signaling pathways and regulate each other to maintain cellular homeostasis [14]. In response to various stress signals, tumor suppressor protein p53 acts as a potent inducer of apoptosis and can also induce autophagy. Autophagy was found possibly to stop cells from undergoing apoptosis by preventing mitochondrial outer membrane permeabilization (MOMP) via blocking the activation of Bax [16, 20]. Upregulation of autophagy in growth factor withdrawal cells can allow cell survival [21] by inhibiting apoptosis. To sum up, autophagy is crucial to cell fates and can protect cells from apoptotic stimuli.

When adding autophagy inhibitor to cMGT cells treated with *E. royleana,* apoptosis sensitivity was increased and therefore caused a large amount of cell death. A clearer link between autophagy and apoptosis has been reported recently. Autophagy imbalance contributes to high autophagy-regulating transcription factor FOXO3a, resulting in the stimulation of the proapoptotic BBC3/PUMA (Bcl2-binding component 3/p53 upregulated modulator of apoptosis) gene to cause apoptosis sensitization [22].

The combination of autophagy inhibitor with different anticancer drugs can increase the likelihood of cancer cell death [23]. At present, chloroquine (CQ) and hydroxychloroquine are the only autophagy inhibitors available in clinical practice in human medicine. By deacidifying the lysosome and blocking the fusion of autophagosomes, these drugs inhibit tumor cell growth or induce tumor cell death [13]. Clinical evidence of glioblastoma treatment involving autophagy inhibitor has received considerable attention recently. Treatment with CQ in conjunction with radiation therapy and the alkylating agent temozolomide demonstrated a statistically significant prolonged median survival time compared with control patient groups [24]. Although the efficacy may vary with different cancer types, a systematic review and meta-analysis indicated that autophagy-inhibitor-based therapy has the optimal treatment response and may provide a new strategy for the treatment of cancer [25]. Thus, the application of autophagy-targeted therapy appears to be a feasible clinical strategy in appropriately selected patient populations. We have recognized that treating cMGT cells with a combination of extracts of *E. royleana* and the autophagy inhibitor Baf can significantly enhance tumor cell death. Based on this evidence, further investigation uncovering the mechanism of autophagy in cMGTs could offer a promising future for new cancer treatment.

## Conclusions

This study clarifies the antitumor effect of *E. royleana* on cMGTs in vitro and in vivo. Blocking autophagy was also found to further increase cell death by triggering apoptosis in *E. royleana*-treated tumors. We hope further studies can be conducted to evaluate the clinical applications of *E. royleana* extracts and autophagy inhibitors for canine mammary tumors.

## Methods

### *E. royleana* extracts and cell culture

The ethanol extract of *E. royleana* (X.E.E.) and hexane extract of *E. royleana* (X.H.E.) were kindly provided by Dr. Tsun-Yung Kuo’s laboratory (National Ilan University, Taiwan). Marc145, and cMGT cell lines, CMT1 [26] and MPG [27] were kindly provided by Dr. Chung-Tien Lin’s laboratory (School of Veterinary Medicine, National Taiwan University). MDCK, Vero, and Marc145 cells were purchased from the Bioresource Collection and Research Center in Taiwan. All cell lines were cultured with Dulbecco’s Modified Eagle’s Medium (DMEM) containing 10% fetal bovine serum (FBS) (Thermo-Fisher Scientific, USA) and 1% antibiotic–antimycotic solution (Roche, Germany) in a humidified incubator with 5% CO_2_ at 37 °C.

### Cell viability and LDH cytotoxic assay

Cells (3 × 10^3^) were treated with the indicated concentration of X.E.E. or X.H.E. for various periods. The cell viability was determined through WST1 assay (Roche, Germany) after the treatment. The effect of the X.E.E. and X.H.E. on the viabilities of cMGT cells were expressed as the cell viability using the formula: % of control cells = [(OD of test samples − OD of blanks)] / [(OD of control samples − OD of blanks)] × 100%. CytoTox 96 nonradioactive cytotoxicity assay (Promega, USA) was used to measure the cytotoxic effects of X.E.E. and X.H.E. according to the manufacturer’s instructions. The amount of lactate dehydrogenase (LDH) release reflects the percentage of cells that underwent necrosis.

### Trypan blue exclusion

Cells (2 × 10^5^) were seeded in the 6-well plate for overnight and then treated with various concentrations of X.E.E. and X.H.E. for different intervals. This was followed by staining with trypan blue dye. The dead cells were counted using a light microscope and calculated with the formula: % of dead cells = [(total dead cells / total live and dead cells)] × 100%.

### Soft agar assay

Cells (2.5 × 10^3^) were seeded in 0.35% agar (Sigma-Aldrich, USA) on top of 0.7% bottom agar in a 24-well plate containing X.E.E. or X.H.E. where indicate. Once a week the plates were overlaid with 0.5 ml low- (0.5% FBS) and high- (5% FBS) serum medium containing X.E.E. or X.H.E. with the indicated concentration. Colonies were counted after 14 days by a light microscope.

### Apoptosis analysis

Annexin V–fluorescein isothiocyanate (FITC) apoptosis detection (Strong Biotech, Taiwan) and sub-G1 analysis were performed by flow cytometry. Cells (4 × 10^5^) were treated with the indicated concentration of X.E.E. or X.H.E. for various periods and using mitomycin C (5 μg/mL) as a positive control. Both early apoptotic (annexin V-positive, propidium iodide (PI)-negative) and late apoptotic (annexin V-positive and PI-positive) cells were included in cell death determinations. For sub-G1 analysis, after X.E.E. or X.H.E. treatment, the cells were fixed and stained with propidium iodide utilizing the PI/RNase Staining Buffer (BD Biosciences, USA) according to the manufacturer’s instructions.

### Autophagosome staining with acridine orange

Acridine orange (AO) cell staining was performed according to the published procedures [28]. Cells (2 × 10^5^) were treated with the indicated concentration of X.E.E. or X.H.E. for 48 h. Then the cells were harvested and stained with 2 μg/mL AO for (Sigma-Aldrich, USA) 5 min incubation. The red or green emitted fluorescence were observed or measured by the fluorescence microscope and flow cytometer.

### Western blotting

The cell lysates treated with X.E.E. or X.H.E. at the indicated concentrations for various periods of time were prepared for immunoblotting of LC3, p53, Caspase 3, Bcl2, Bcl2-associated X protein (Genetex, USA). Western blot analysis was performed as previously reported [29]. The original blots can be referred in the additional file 1.

### Blocking autophagy

Cells (3 × 10^3^) were precultured with different concentrations (1–9 nM) of autophagy inhibitor, bafilomycin A1 (Baf) (Sigma-Aldrich, USA) for 2 h; subsequently the medium was changed with 10 μg/mL X.E.E. and 3 μg/mL X.H.E., and the cells were incubated for 3 days. Cell viability was determined after 3 days through WST1 assay.

### cMGT xenograft model

Male NCr athymic nude mice aged 8–10 weeks were obtained from National Laboratory Animal Center (Taipei, Taiwan). The mice were maintained in accordance with protocols approved by the Institutional Animal Care and Use Committee of National Taiwan University (IACUC No. NTU-100-EL-90). 5 × 10^6^ cells were inoculated into each mouse. Tumor volume based on caliper measurements were calculated by the modified ellipsoidal formula: Tumor volume = 1/2 (length × width^2^). When CMT1 cells were inoculated subcutaneously and the tumor is reached 300–500 mm^3^, mice (*N* = 6 in each treatment) were randomized distributed in each group and received X.H.E. (10 mg/kg) p.o. every 2–4 days. Controls received vehicle. The duration of the treatment was 5 weeks and then the animals were euthanized by CO_2_ overdose exposure. The tumor samples were collected after the treatment. Three independent experiments were conducted and the significance was analyzed in each individual test.

### Statistical analysis

Results were expressed as mean ± standard deviation. The data were analyzed by a two- way ANOVA followed by post-hoc Tukey HSD test (GRAPHPAD PRISM, GraphPad Software Inc., USA). Differences with *p*-values *p < 0.05, **p < 0.01 were considered statistically significant.

## Supplementary information

**Additional file 1.** The original blots for the figures.

## Data Availability

The datasets used and/or analyzed during the current study are available from the corresponding author on reasonable request.

## Abbreviations

AO: Acridine orange
BBC3: Bcl-2-binding component 3
Bcl2: B-cell lymphoma 2
Baf: Bafilomycin A1
Bax: Bcl2-Associated X Protein
CQ: Chloroquine
cMGTs: Canine mammary gland tumors
*Euphorbia royleana*: *E. royleana*
FBS: Fetal bovine serum
FITC: Fluorescein isothiocyanate
FOXO3a: Forkhead box O3
IC_50_: The half maximal inhibitory concentration
LC3: Microtubule-associated light chain 3
LDH: Lactate dehydrogenase
MOMP: Mitochondrial outer membrane permeabilization
PI: Propidium iodide
PUMA: p53 upregulated modulator of apoptosis
X.E.E.: Ethanol extract of *E. royleana*
X.H.E.: Hexane extract of *E. royleana*
