# Supplementary material for: Inhibiting autophagy potentiates the antitumor efficacy of Euphorbia royleana for canine mammary gland tumors
Source: BMC Vet Res. 2020 Jun 12;16:193. doi: 10.1186/s12917-020-02408-1 (PMC7291717; doi:10.1186/s12917-020-02408-1)

**The original blots for the figures**

**Note:** For our gel data, the target proteins analyzed by western blot were transferred from SDS-PAGE and the PVDF membrane would be sliced into different strips according to their protein size identified with the loading marker. Then the panel of antibodies targeting these proteins were used for western blot analysis. Our SOP for processing the gel data only collected the pictures with target protein regions instead of the whole gel, so here are the original gels for the data we presented in the manuscript.

**Fig. 5B~Caspase 3**


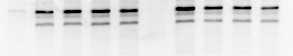

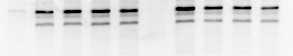


| EtOH | X.E.E. | | |
| --- | --- | --- | --- |
|  | 10 | 15 | 20 |

| EtOH | X.H.E. | | |
| --- | --- | --- | --- |
|  | 3 | 5 | 10 |

**Fig. 5B~ Bcl2**

**Fig. 5B ~Bcl2**

| EtOH | X.E.E. | | |
| --- | --- | --- | --- |
|  | 10 | 15 | 20 |

| EtOH | X.H.E. | | |
| --- | --- | --- | --- |
|  | 3 | 5 | 10 |


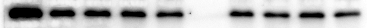


**Fig. 5B~ Bax**


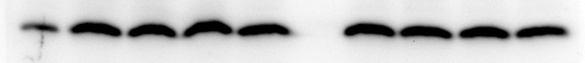


| EtOH | X.E.E. | | |
| --- | --- | --- | --- |
|  | 10 | 15 | 20 |

| EtOH | X.H.E. | | |
| --- | --- | --- | --- |
|  | 3 | 5 | 10 |


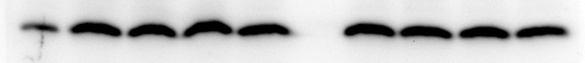


| EtOH | X.H.E. | | |
| --- | --- | --- | --- |
|  | 3 | 5 | 10 |

**Fig. 5B~ α-actin**

| EtOH | X.E.E. | | |
| --- | --- | --- | --- |
|  | 10 | 15 | 20 |


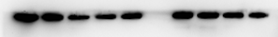


**Fig. 6E~P53**

**MPG**

| EtOH | X.E.E.  20 | DMSO | X.H.E.  10 |
| --- | --- | --- | --- |


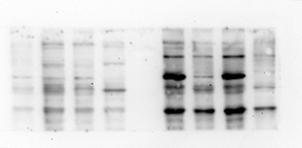


**CMT**

| EtOH | X.E.E.  20 | DMSO | X.H.E.  10 |
| --- | --- | --- | --- |


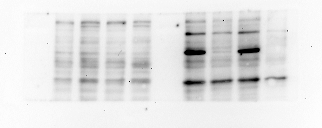


**Fig. 6E~LC-I &II**

**MPG**

| EtOH | X.E.E.  20 | DMSO | X.H.E.  10 |
| --- | --- | --- | --- |


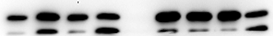


**CMT**

| EtOH | X.E.E.  20 | DMSO | X.H.E.  10 |
| --- | --- | --- | --- |


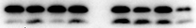


**Fig. 6E~ α-actin**

**MPG**

| EtOH | X.E.E.  20 | DMSO | X.H.E.  10 |
| --- | --- | --- | --- |


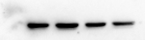


**CMT**

| EtOH | X.E.E.  20 | DMSO | X.H.E.  10 |
| --- | --- | --- | --- |


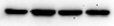


**Fig. 7A ~ LC-I &II (MPG)**

| Baf A1 | MPG | |
| --- | --- | --- |
|  | **+** | **-** |


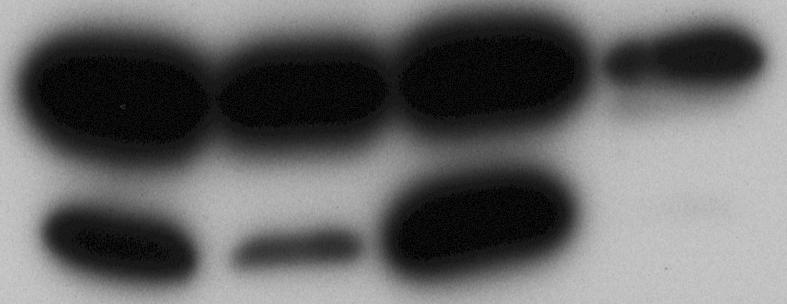


| Baf A1 | MPG | |
| --- | --- | --- |
|  | **+** | **-** |

**Fig. 7A ~ α-actin (MPG)**


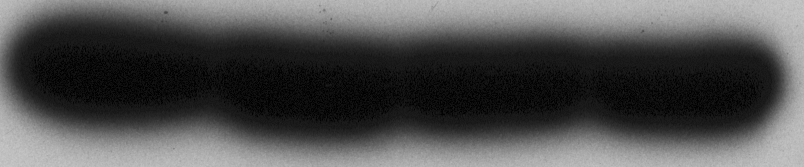


**Fig. 7A ~ LC-I &II (CMT-1)**

| Baf A1 | CMT-1 | |
| --- | --- | --- |
|  | **+** | **-** |


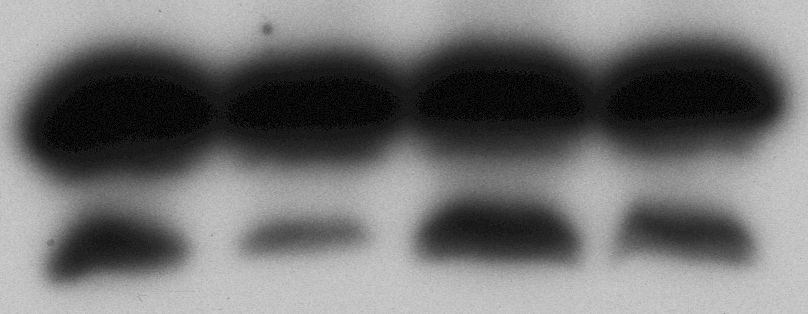


**Fig. 7A ~ α-actin (CMT-1)**

| Baf A1 | CMT-1 | |
| --- | --- | --- |
|  | **+** | **-** |


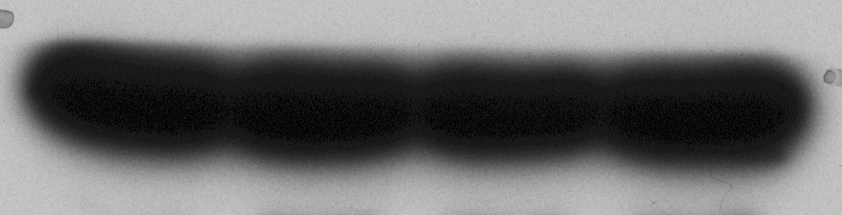

Supplement: Supplementary file 1 — Additional file 1. The original blots for the figures. [file 12917_2020_2408_MOESM1_ESM.docx]
